# Supplementary material for: Networks analysis of Brazilian climate data based on the DCCA cross-correlation coefficient
Source: PLoS One. 2023 Sep 15;18(9):e0290838. doi: 10.1371/journal.pone.0290838 (PMC10503753; doi:10.1371/journal.pone.0290838)
Supplement: S1 File — (PDF) [file pone.0290838.s001.pdf]

Networks Analysis of Brazilian Climate Data based on the DCCA cross-correlation coefficient

Florêncio Mendes Oliveira Filho[1,2*], Everaldo Freitas Guedes[3], and Paulo Canas Rodrigues[4]

[1]Senai Cimatec University Center, Computer Engineering, Salvador, Brazil
[2]Earth Sciences and Environment Modeling Program, State University of Feira de Santana, Feira de Santana, BA, Brazil
[3]Brazilian Hospital Services Company - Climério de Oliveira Maternity Hospital, Health Regulation and Evaluation Sector, Salvador, Brazil
[4]Federal University of Bahia, Department of Statistics, Salvador, Brazil

1
Brazilian regions and states

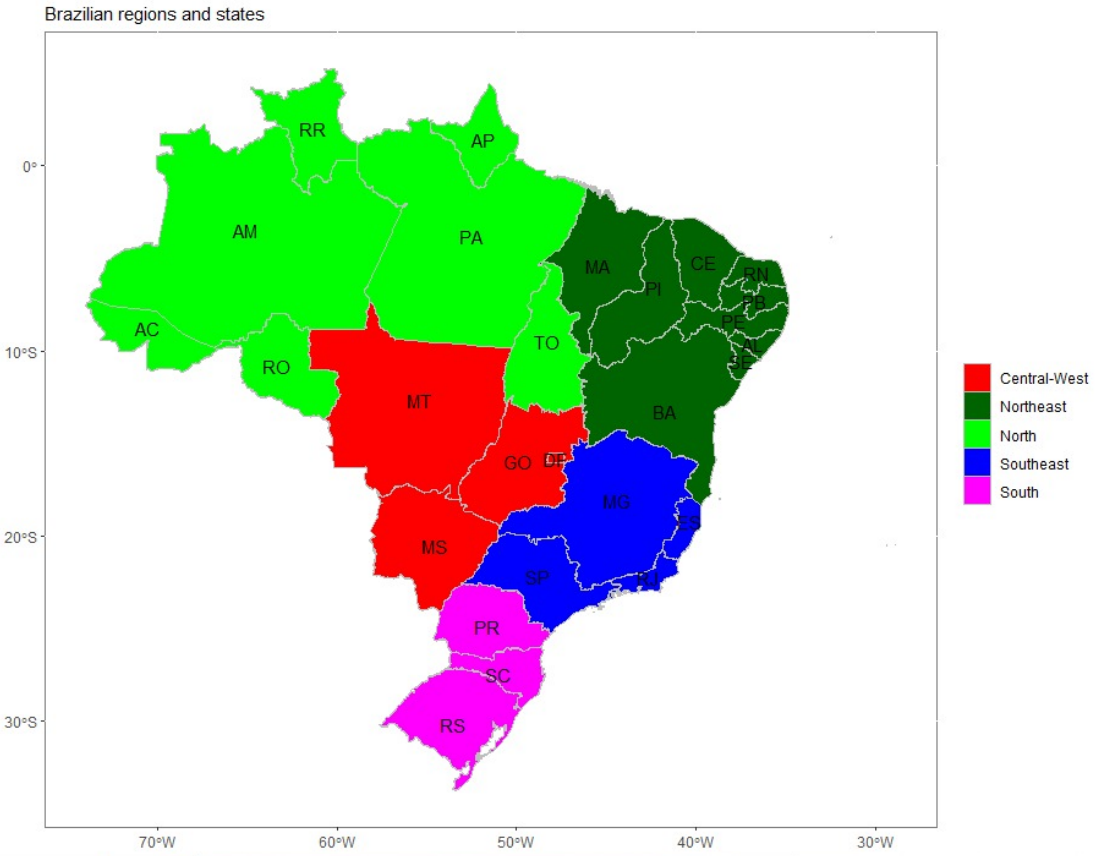

Figure 1S: Map of Brazil with the five regions and the 26 Brazilian state capitals and in the federal district.

2
Detrended fluctuation analysis (DFA)

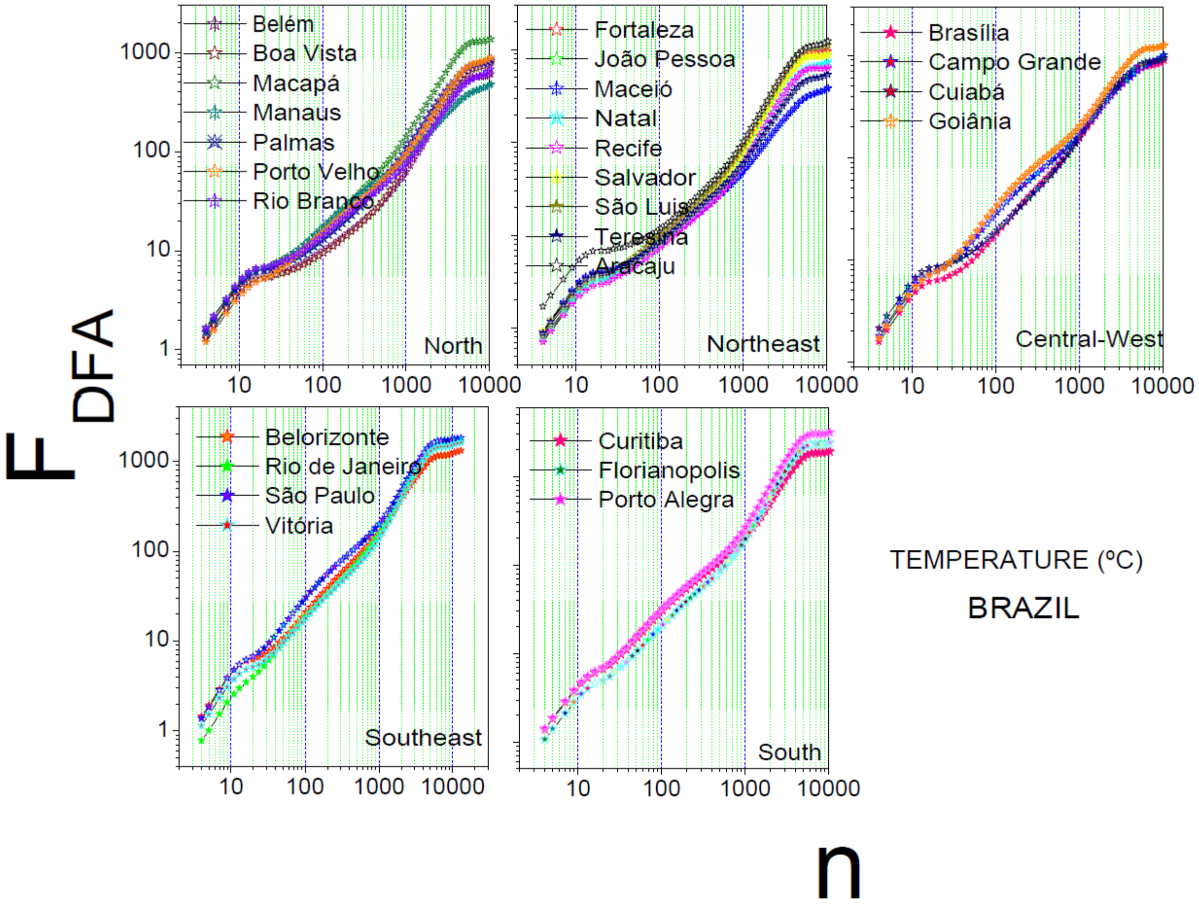

Figure 2S: Detrended fluctuation analysis for temperature. The plots show, respectively the curves for all capitals in the regions North, Northeast, Central-West, Southeast and South, respectively. The vertical axis give the F_{DFA} and the horizontal axis show the box size.

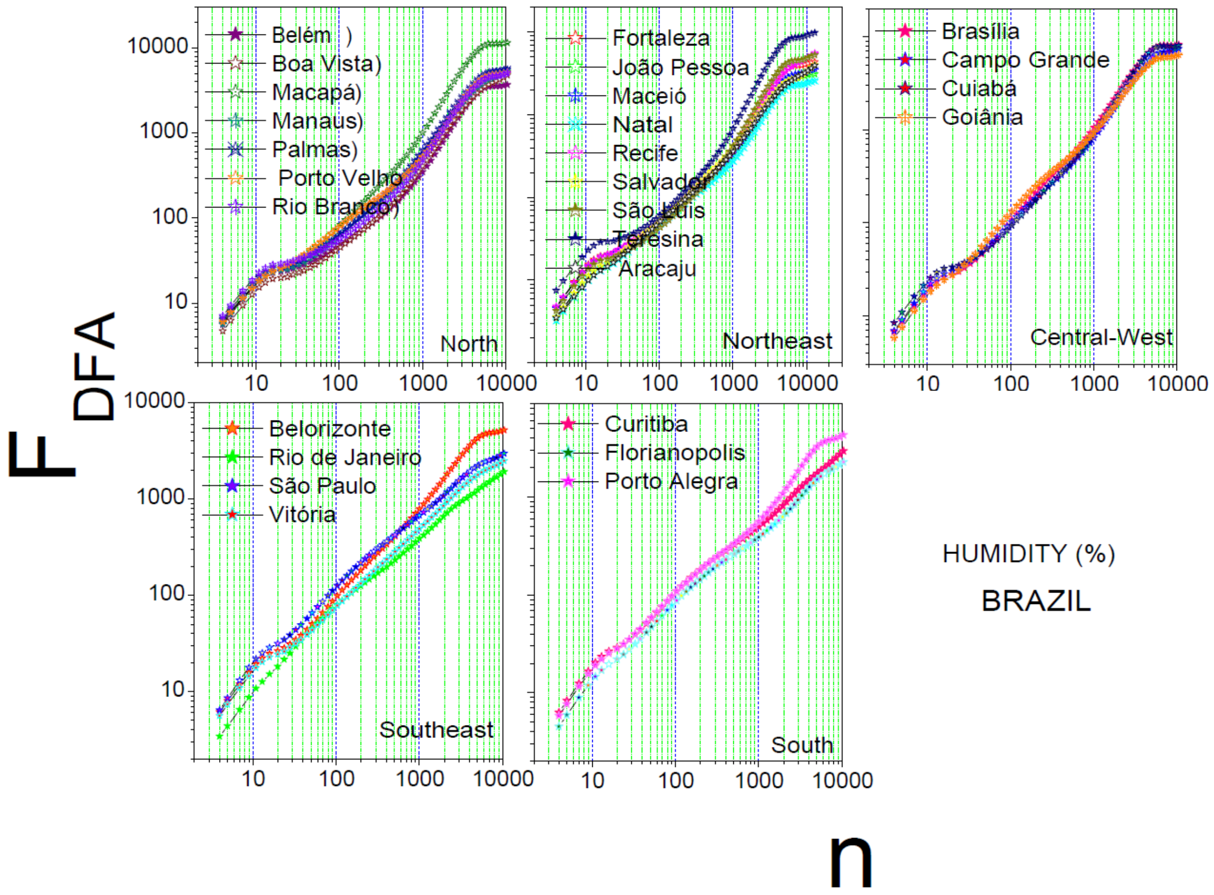

Figure 3S: Detrended fluctuation analysis for humidity. The plots show, respectively the curves for all capitals in the regions North, Northeast, Central-West, Southeast and South, respectively. The vertical axis give the F_{DFA} and the horizontal axis show the box size.

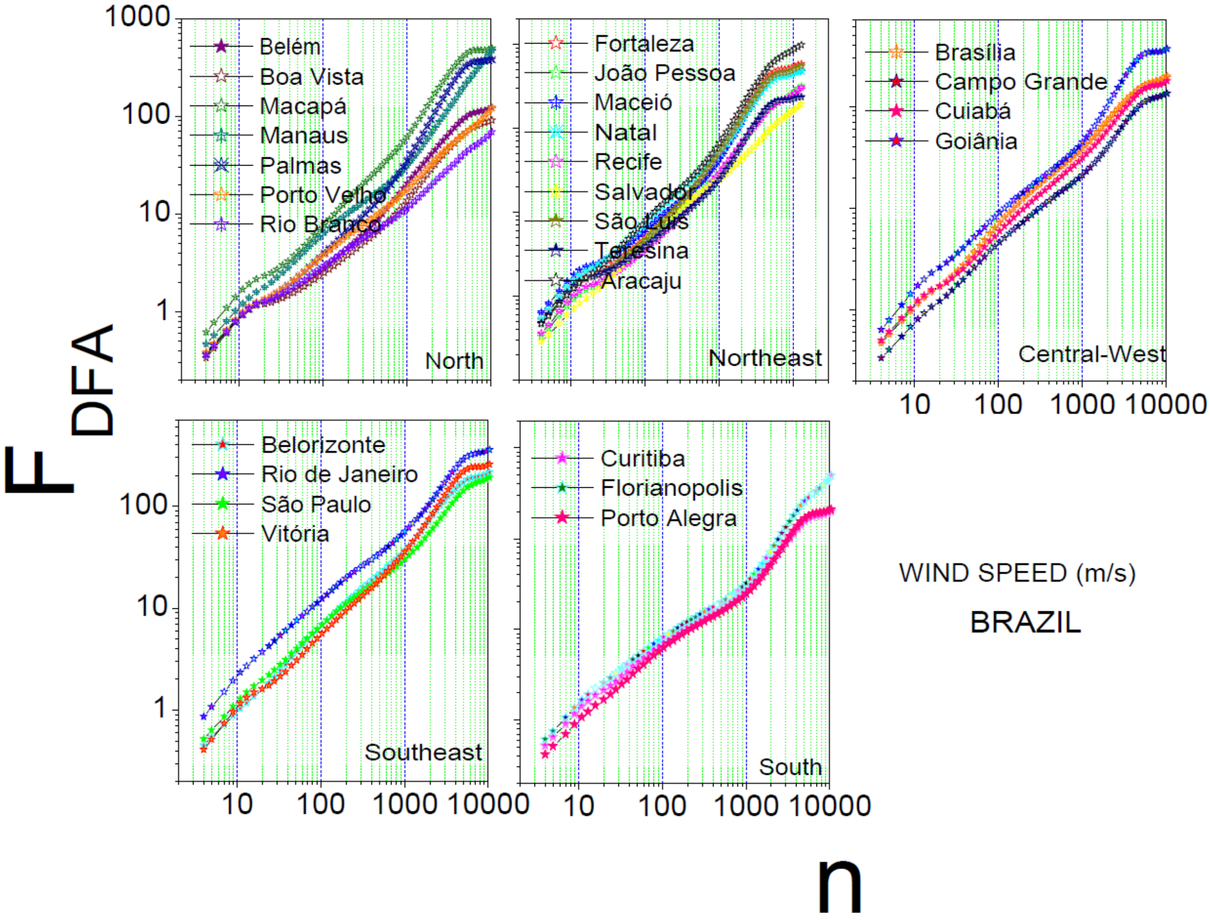

Figure 4S: Detrended fluctuation analysis for wind speed. The plots show, respectively the curves for all capitals in the regions North, Northeast, Central-West, Southeast and South, respectively. The vertical axis give the F_{DFA} and the horizontal axis show the box size.

3

Cross Correlation, $\rho_{DCCA}(n)$, for the North Region

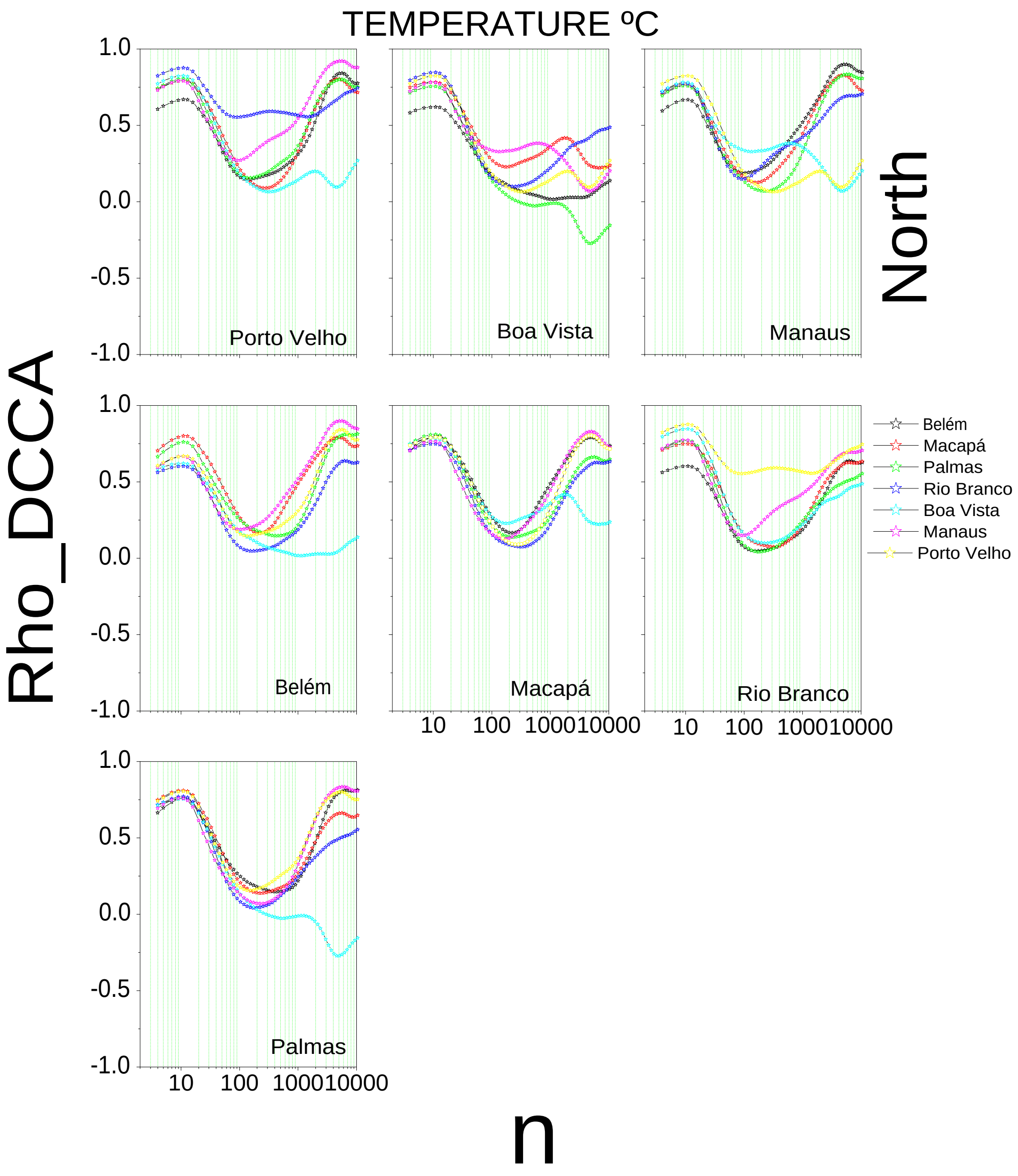

Figure 5S: Cross-correlation, $\rho_{DCCA}(n)$, for temperature in the North region of Brazil. The plots show the cross-correlations between the state capital written in the plot and all others in the region.

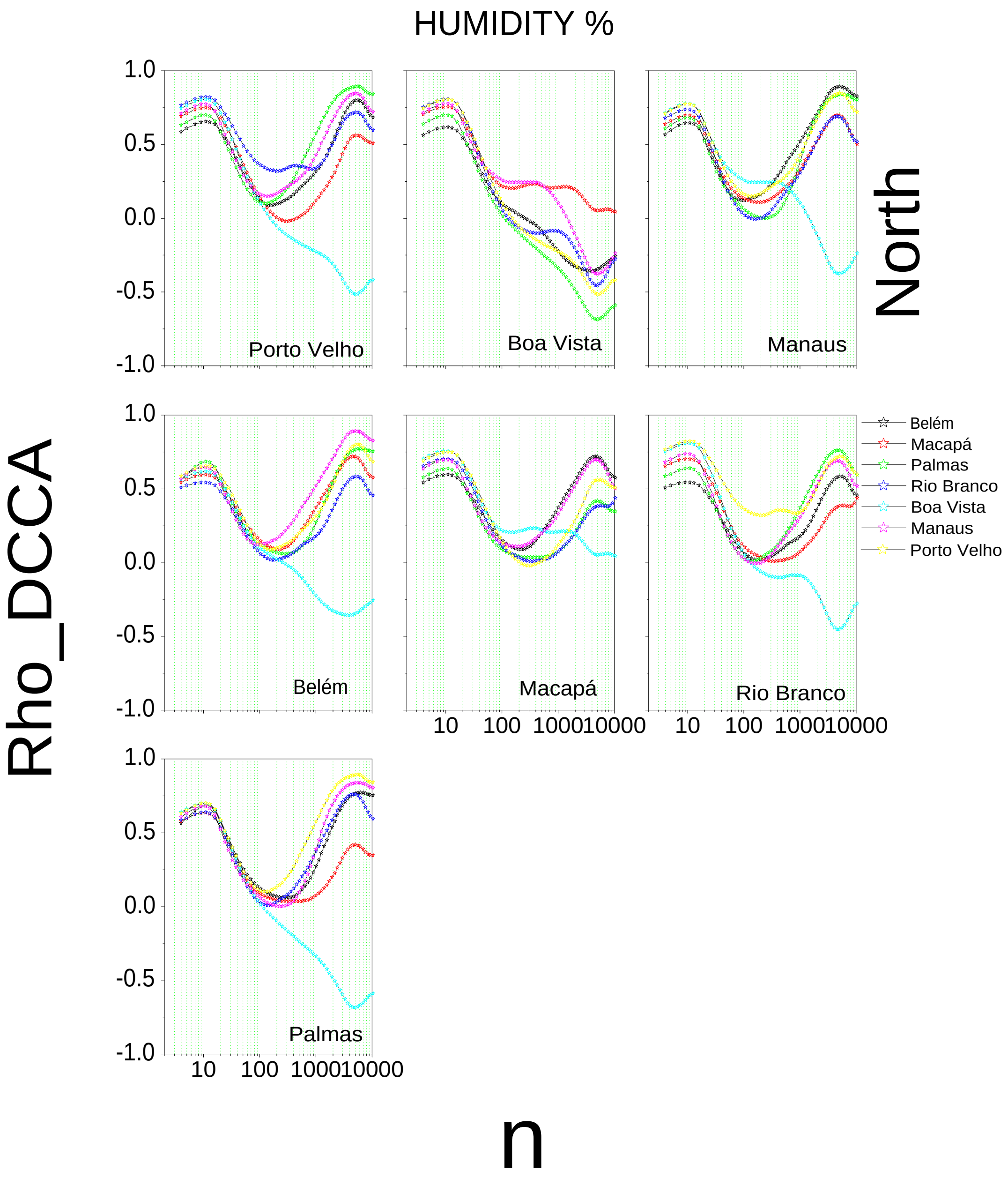

Figure 6S: Cross-correlation, $\rho_{DCCA}(n)$, for humidity in the North region of Brazil. The plots show the cross-correlations between the state capital written in the plot and all others in the region.

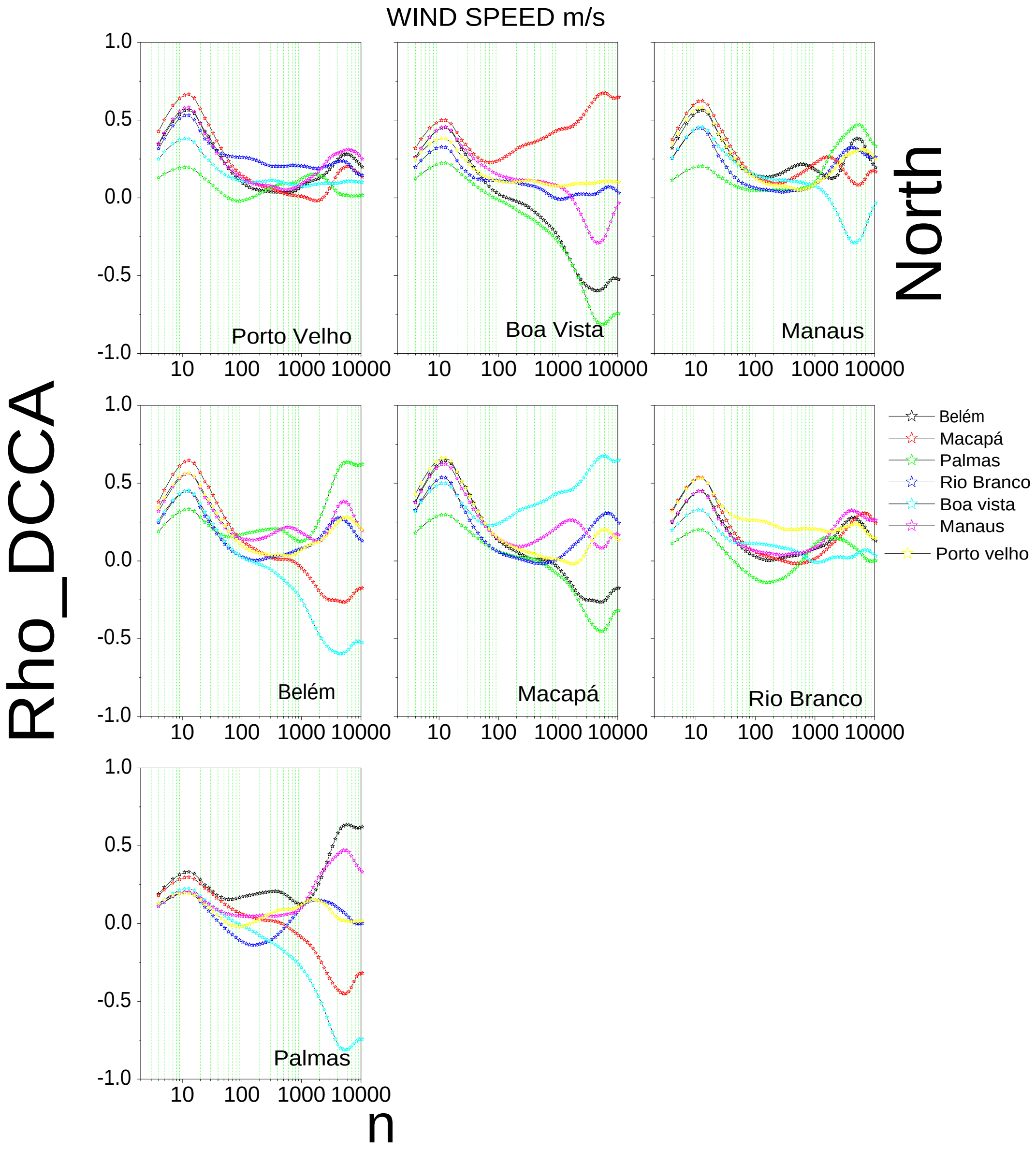

Figure 7S: Cross-correlation, $\rho_{DCCA}(n)$, for wind speed in the North region of Brazil. The plots show the cross-correlations between the state capital written in the plot and all others in the region.

4

Cross Correlation, $\rho_{DCCA}(n)$, for the Northeast Region

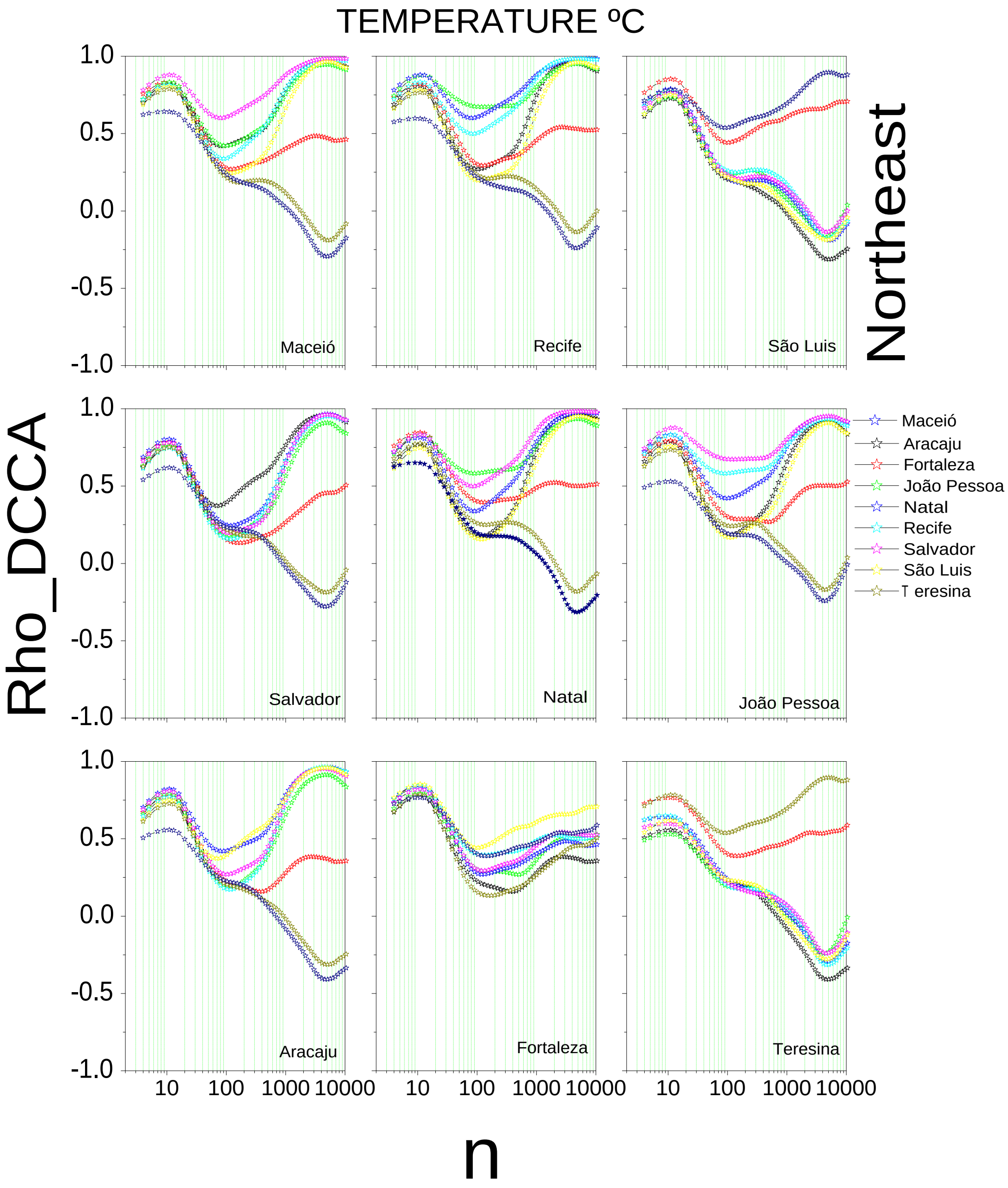

Figure 8S: Cross-correlation, $\rho_{DCCA}(n)$, for temperature in the Northeast region of Brazil. The plots show the cross-correlations between the state capital written in the plot and all others in the region.

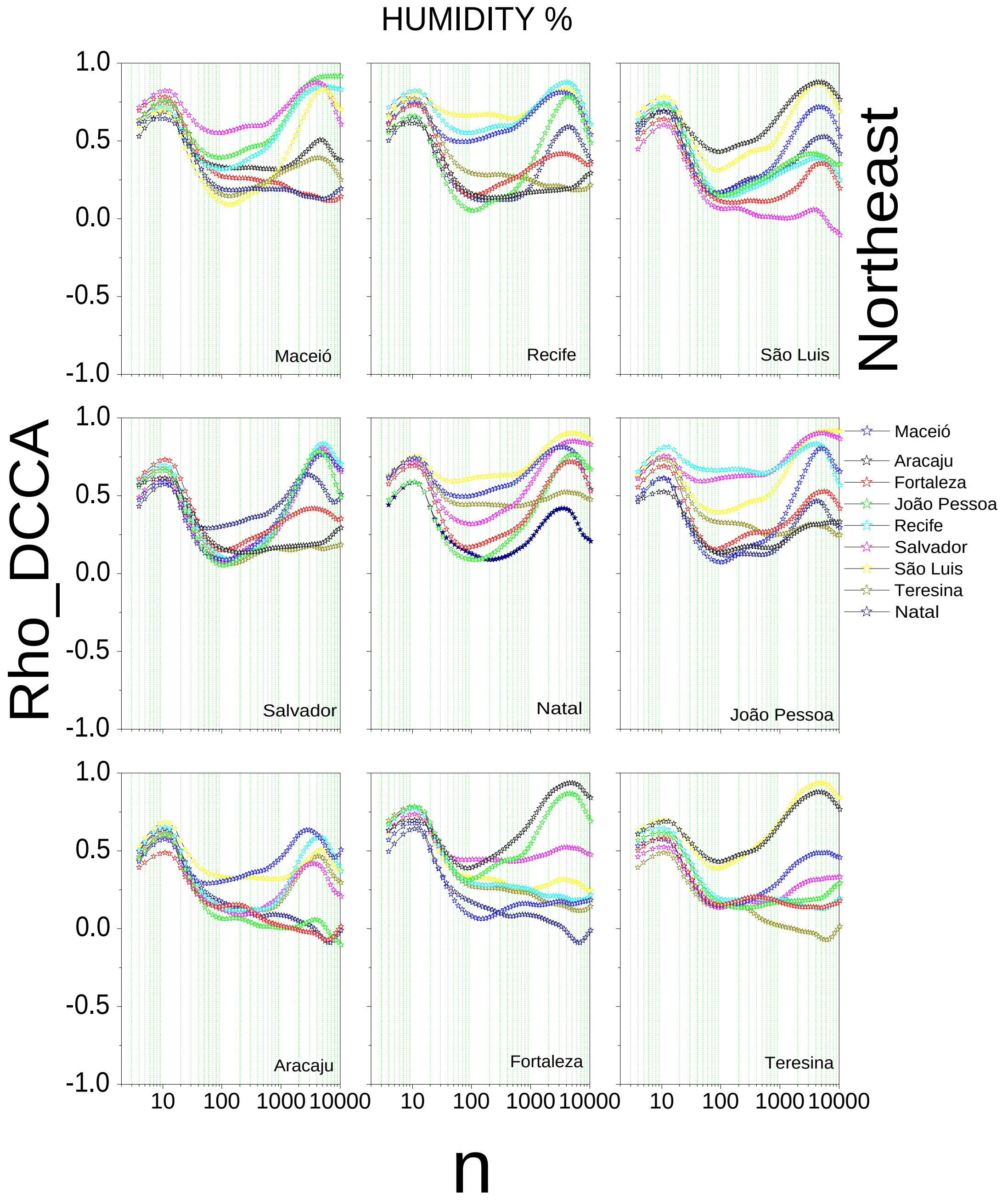

Figure 9S: Cross-correlation, $\rho_{DCCA}(n)$, for humidity in the Northeast region of Brazil. The plots show the cross-correlations between the state capital written in the plot and all others in the region.

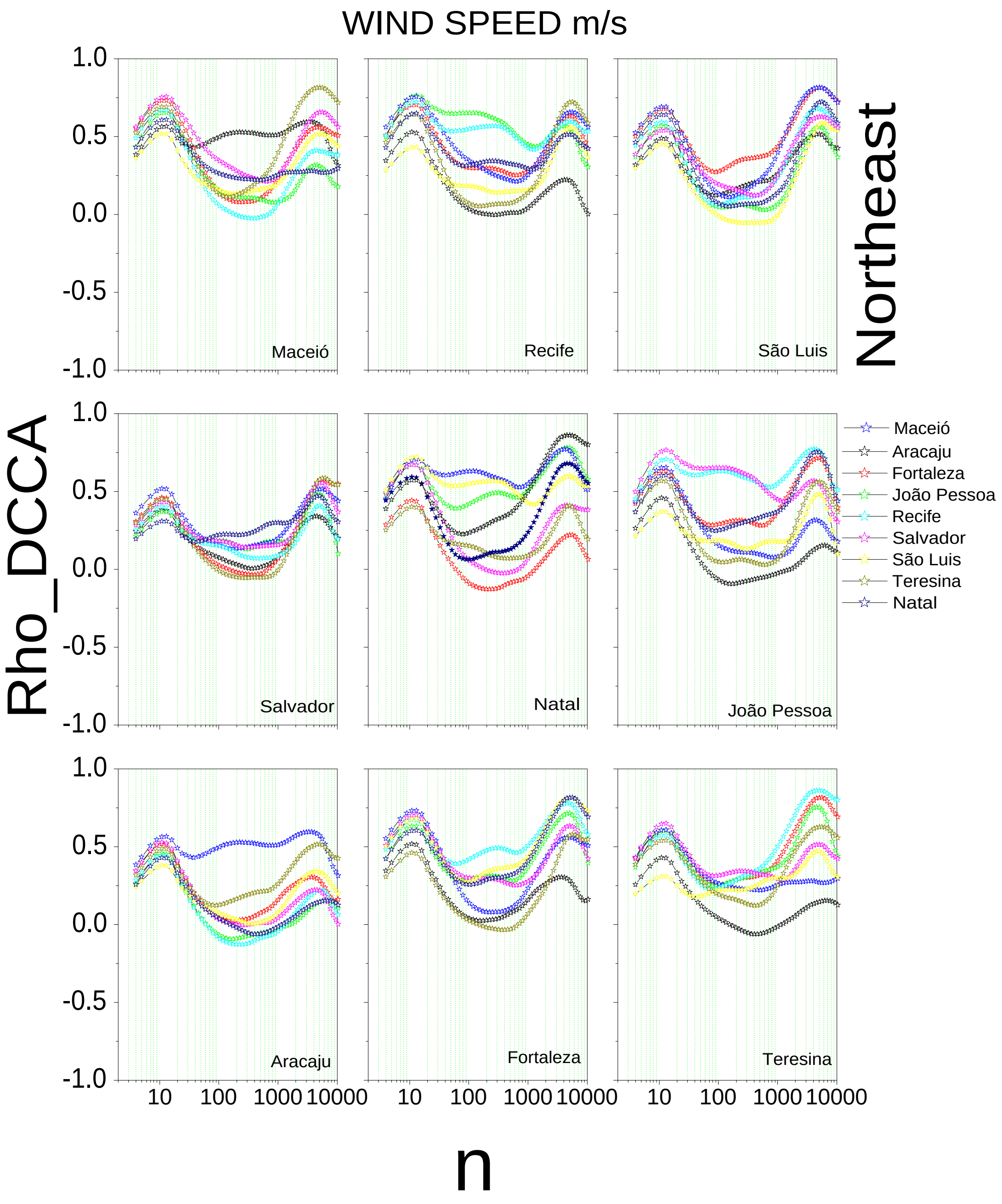

Figure 10S: Cross-correlation, $\rho_{DCCA}(n)$, for wind speed in the Northeast region of Brazil. The plots show the cross-correlations between the state capital written in the plot and all others in the region.

5

Cross Correlation, $\rho_{DCCA}(n)$, for the Central-West Region

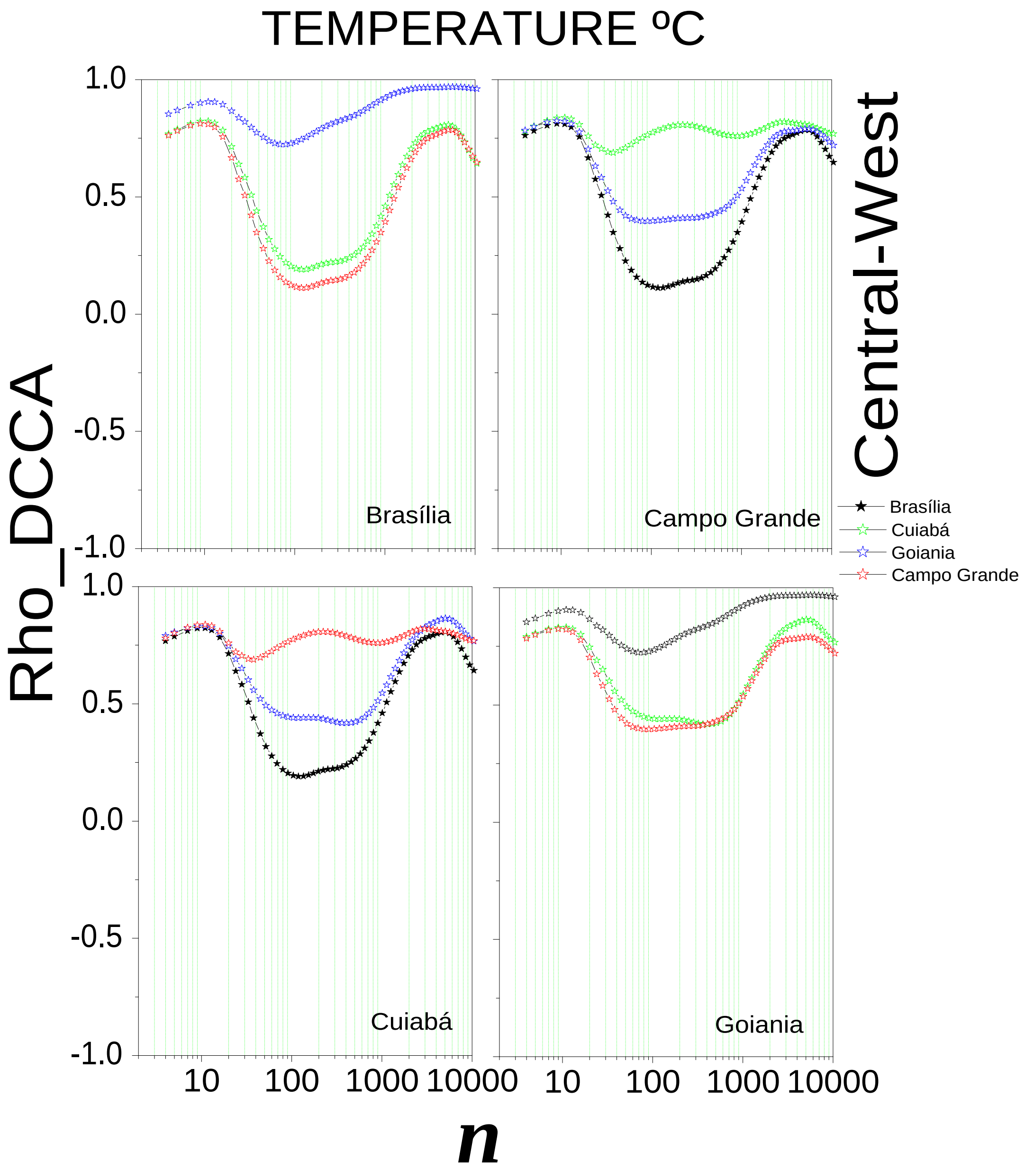

Figure 11S: Cross-correlation, $\rho_{DCCA}(n)$, for temperature in the Central-West region of Brazil. The plots show the cross-correlations between the state capital written in the plot and all others in the region.

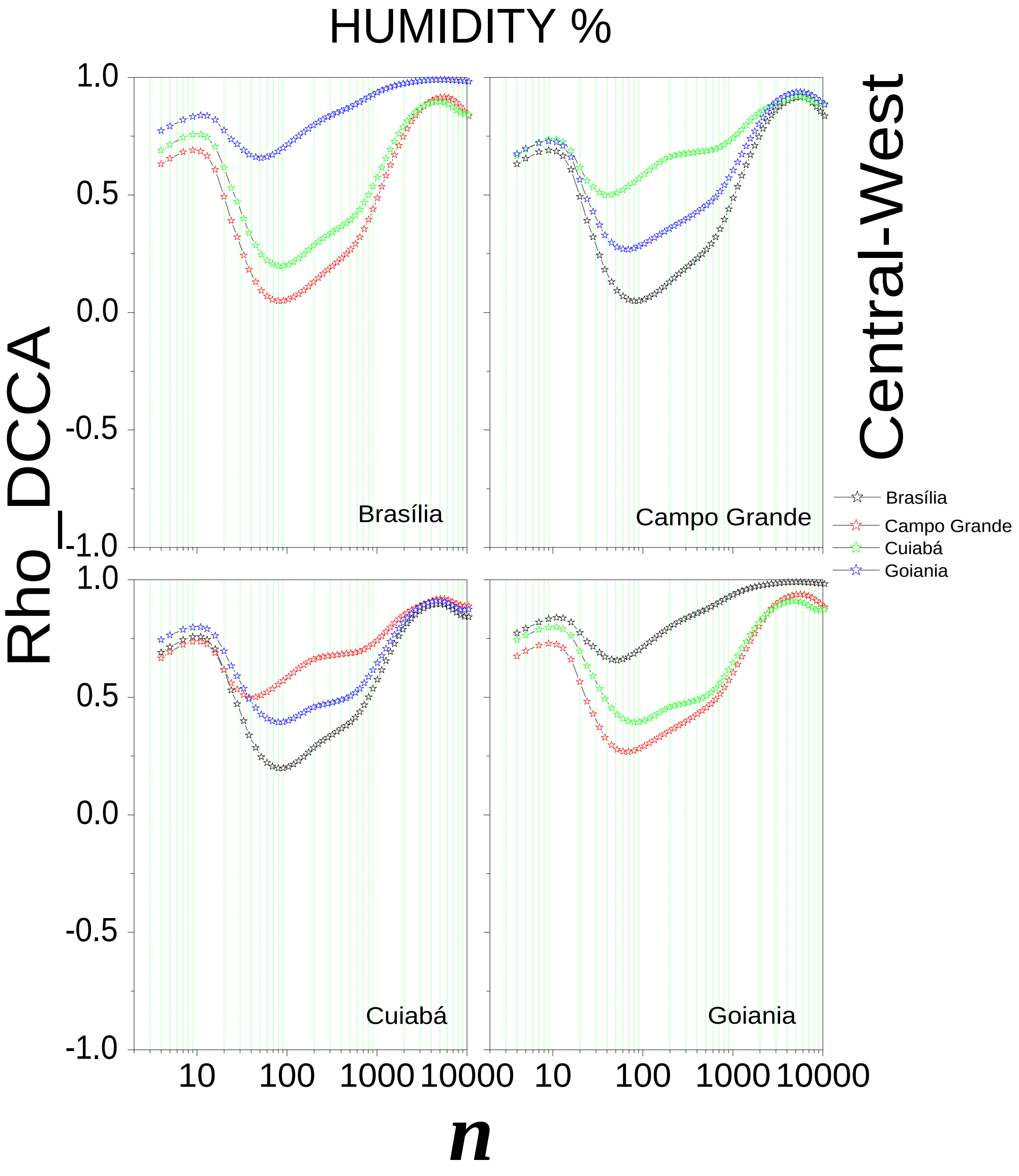

Figure 12S: Cross-correlation, $\rho_{DCCA}(n)$, for humidity in the Central-West region of Brazil. The plots show the cross-correlations between the state capital written in the plot and all others in the region.

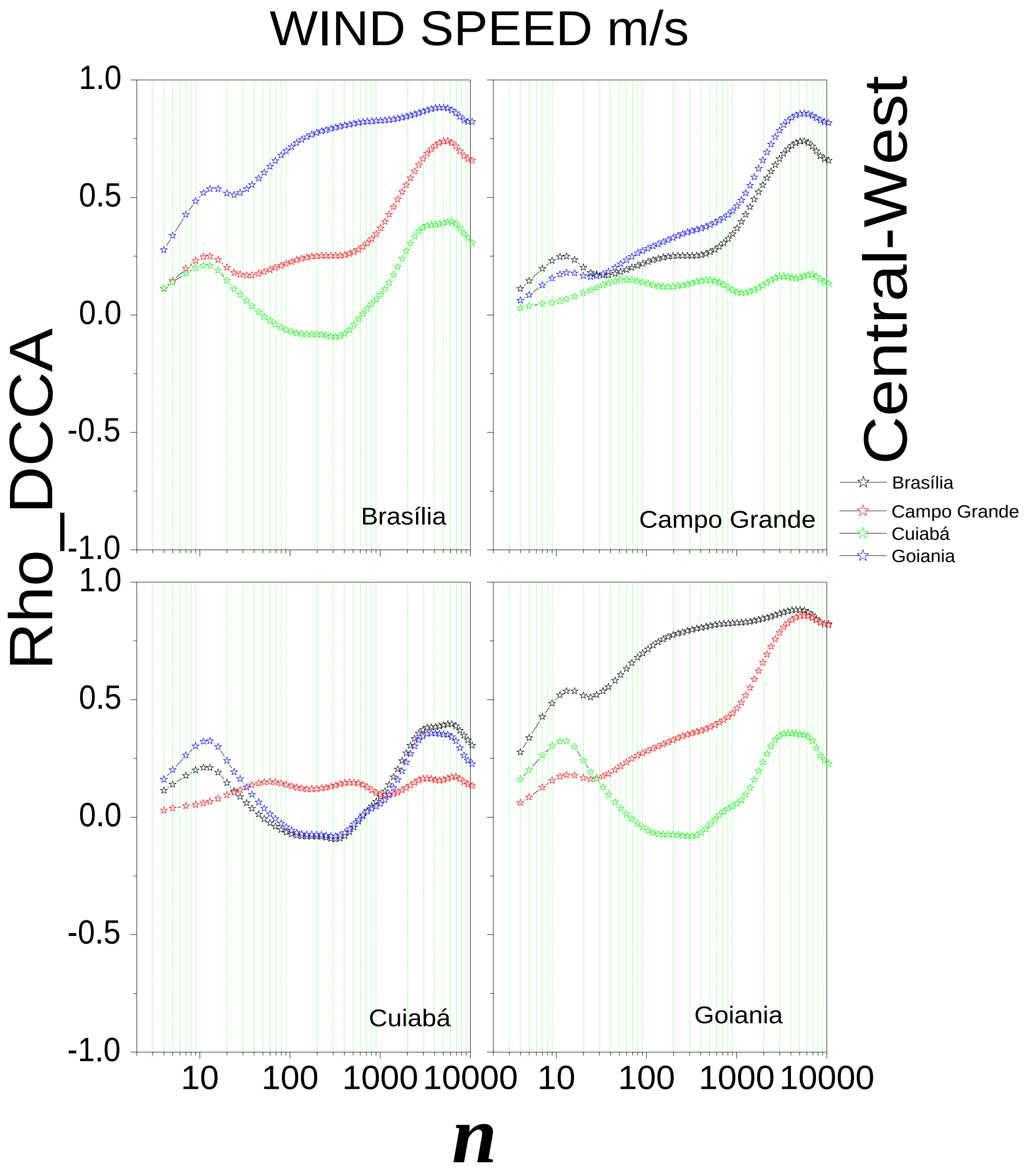

Figure 13S: Cross-correlation, $\rho_{DCCA}(n)$, for wind speed in the Central-West region of Brazil. The plots show the cross-correlations between the state capital written in the plot and all others in the region.

6

Cross Correlation, $\rho_{DCCA}(n)$, for the Southeast Region

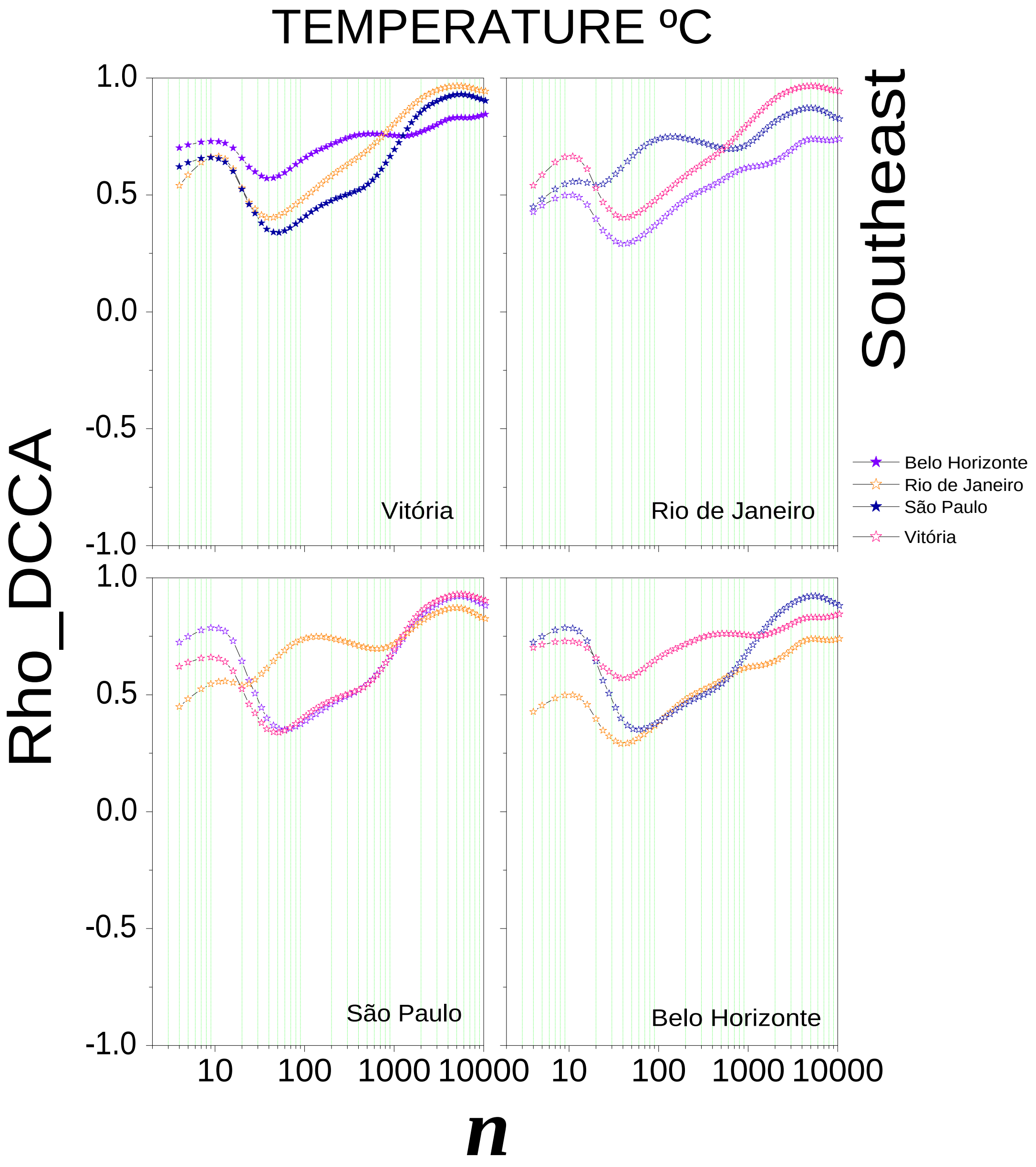

Figure 14S: Cross-correlation, $\rho_{DCCA}(n)$, for temperature in the Southeast region of Brazil. The plots show the cross-correlations between the state capital written in the plot and all others in the region.

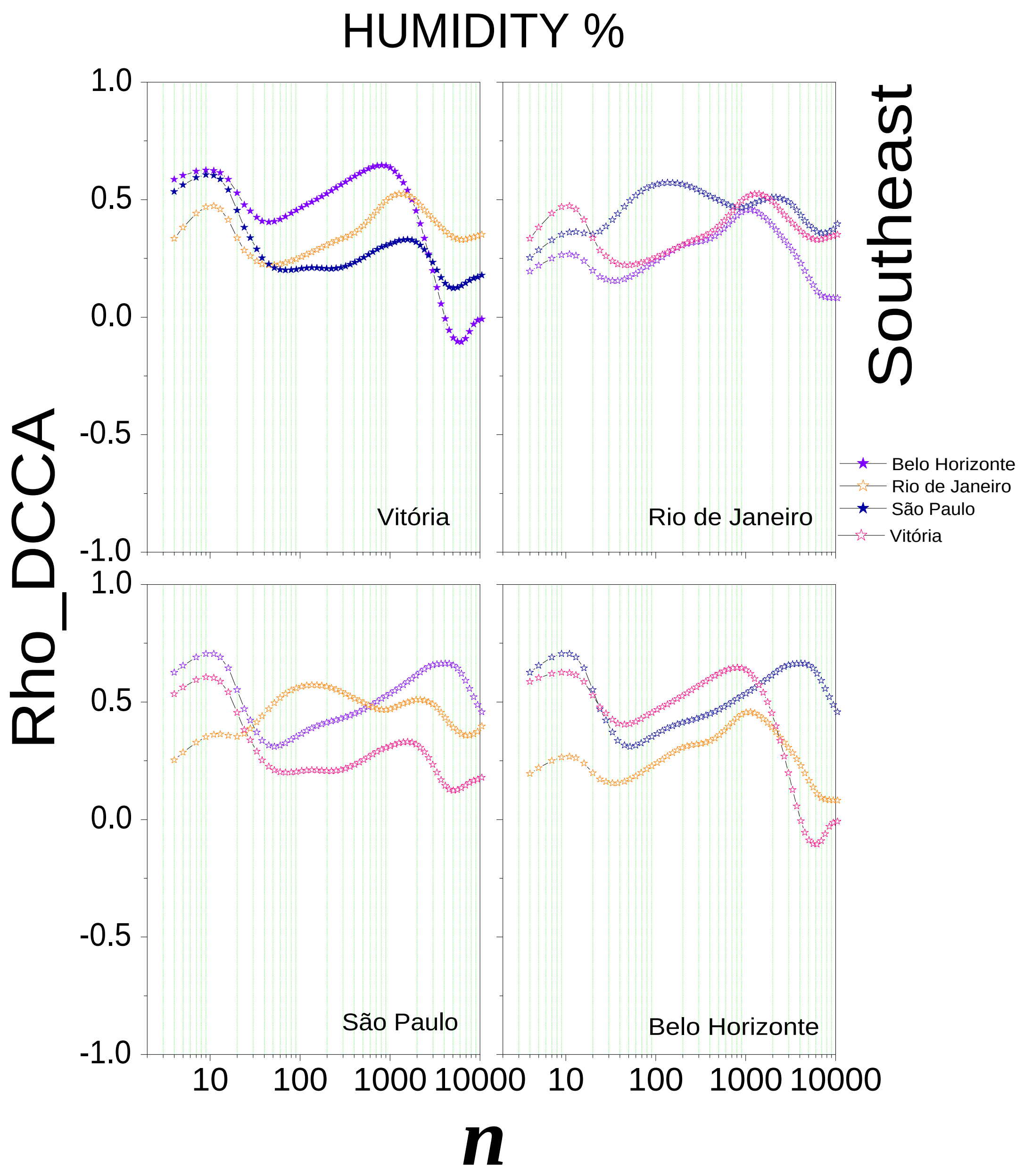

Figure 15S: Cross-correlation, $\rho_{DCCA}(n)$, for humidity in the Southeast region of Brazil. The plots show the cross-correlations between the state capital written in the plot and all others in the region.

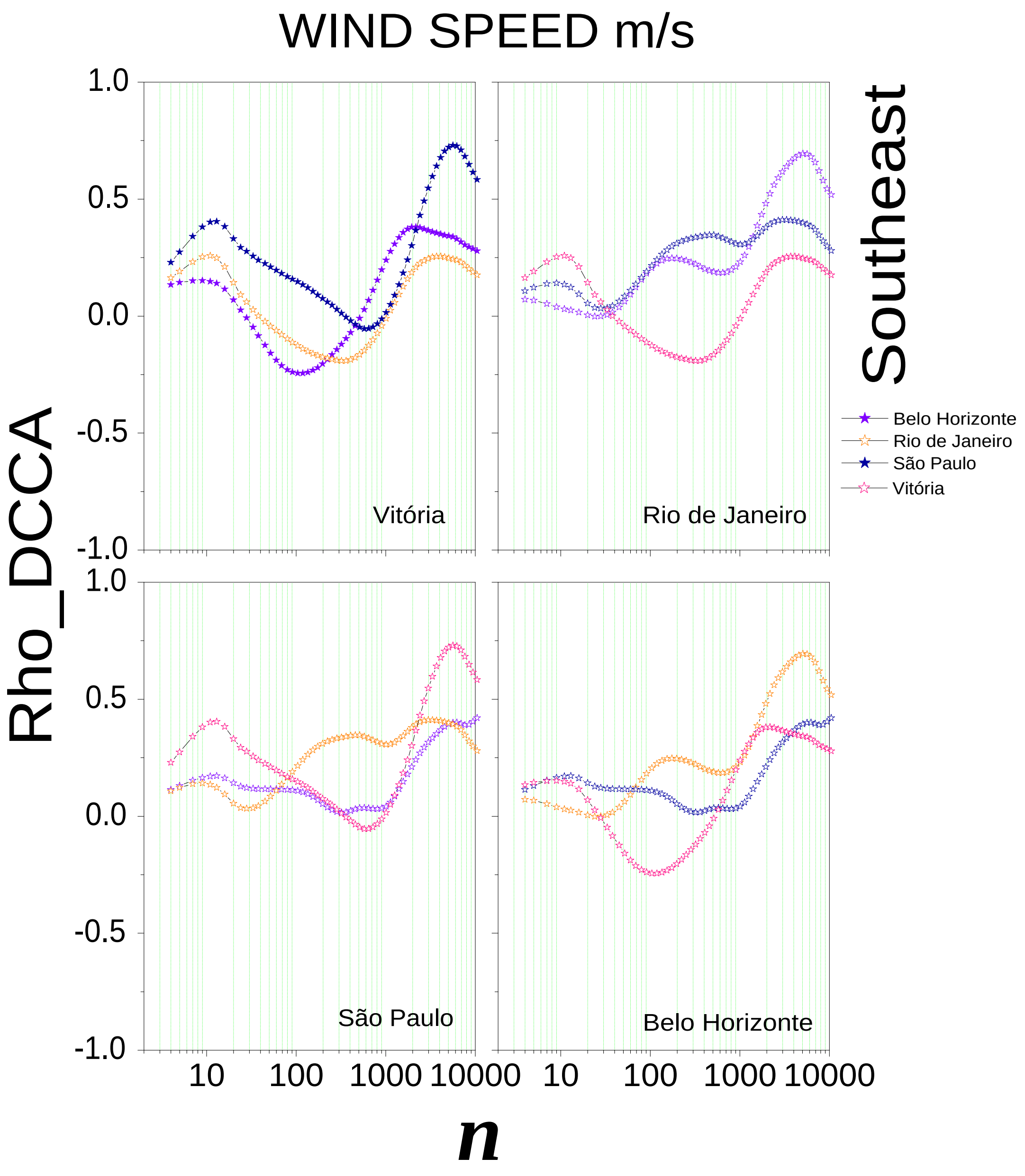

Figure 16S: Cross-correlation, $\rho_{DCCA}(n)$, for wind speed in the Southeast region of Brazil. The plots show the cross-correlations between the state capital written in the plot and all others in the region.

7

Cross Correlation, $\rho_{DCCA}(n)$, for the South Region

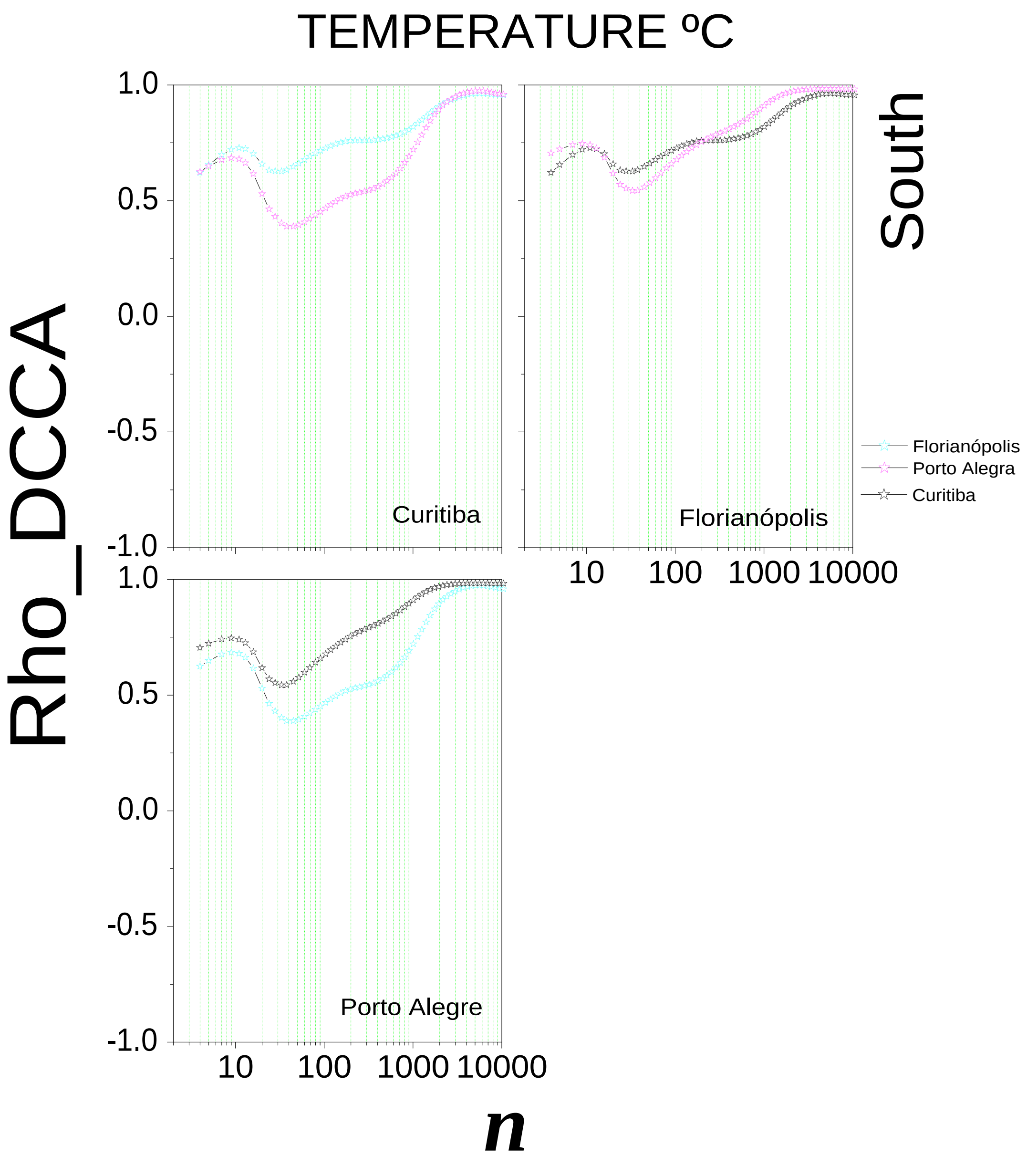

Figure 17S: Cross-correlation, $\rho_{DCCA}(n)$, for temperature in the South region of Brazil. The plots show the cross-correlations between the state capital written in the plot and all others in the region.

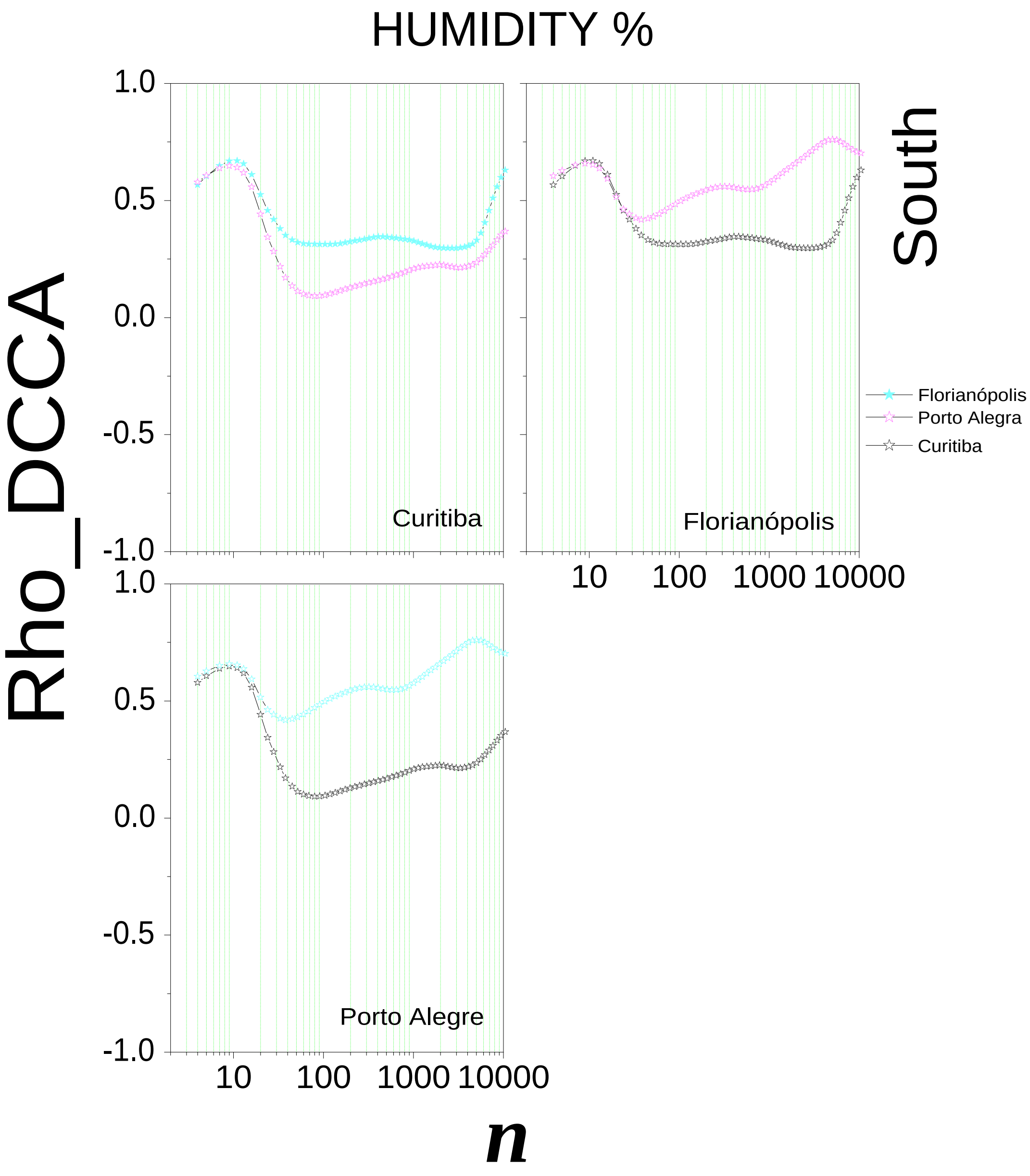

Figure 18S: Cross-correlation, $\rho_{DCCA}(n)$, for humidity in the South region of Brazil. The plots show the cross-correlations between the state capital written in the plot and all others in the region.

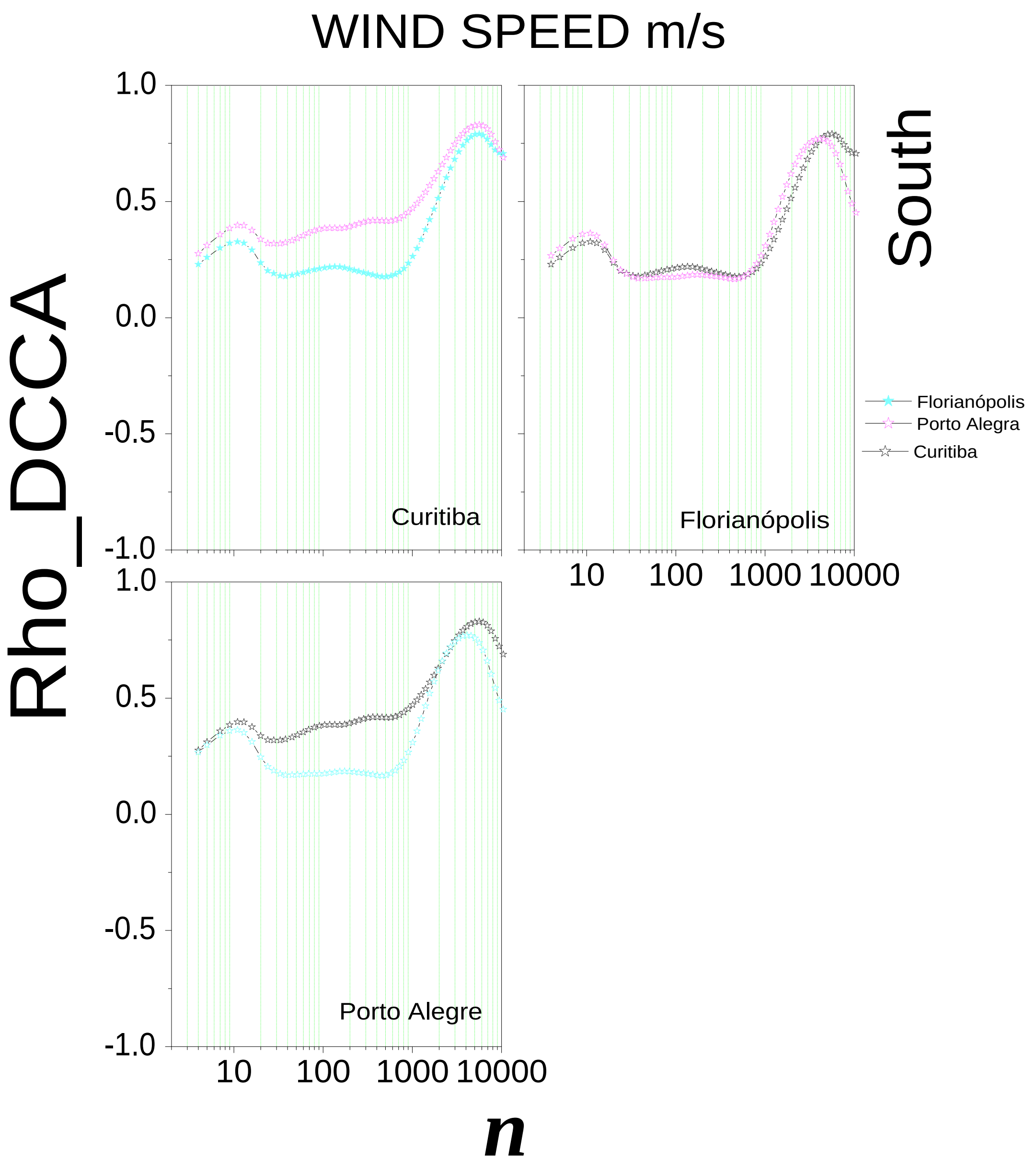

Figure 19S: Cross-correlation, $\rho_{DCCA}(n)$, for wind speed in the South region of Brazil. The plots show the cross-correlations between the state capital written in the plot and all others in the region.

8
Descriptive statistics

Table 1S: Descriptive statistics for global radiation.

Radiação	Cities	Mean	Std. dev.	Skewness	Kurtosis	Minimum	Q1	Median	Q3	Maximum
North	Belém	1158.4	976.9	0.5	2.0	0.0	214.7	967.5	1972.1	5230.0
	Boa Vista	1465.5	1113.3	0.2	1.8	0.0	341.6	1430.7	2462.5	6196.8
	Macapá	1227.8	925.8	0.3	2.0	0.0	330.3	1190.6	1990.6	3993.5
	Manaus	1220.7	1014.1	0.4	1.9	0.0	243.8	1041.6	2059.5	3947.0
	Palmas	1317.6	1066.7	0.3	1.7	0.0	234.3	1192.1	2289.6	3969.7
	Porto Velho	1266.9	1009.6	0.3	1.9	0.0	251.6	1189.2	2125.4	4156.5
	Rio Branco	1284.7	1024.2	0.2	1.8	0.0	213.0	1248.1	2159.2	6682.6
Northeast	Fortaleza	1310.3	926.2	0.1	1.8	0.0	431.4	1297.5	2103.2	4403.2
	João Pessoa	1586.9	1164.9	0.1	1.7	0.0	409.8	1598.5	2631.0	4884.3
	Maceió	1490.2	1123.7	0.3	1.9	0.0	386.4	1425.6	2415.9	4694.6
	Natal	1653.4	1236.2	0.2	1.7	0.1	440.9	1594.3	2761.6	4719.5
	Recife	1606.4	1149.1	0.2	2.0	0.0	494.7	1619.5	2518.4	4898.8
	Salvador	1442.3	1151.6	0.4	1.8	0.0	342.3	1284.6	2418.7	4158.8
	São Luís	1369.3	988.6	0.4	2.4	0.0	359.5	1303.9	2146.6	7850.2
	Teresina	1296.2	919.2	0.2	1.9	0.0	429.7	1295.4	2065.2	3901.7
	Aracaju	1381.0	1018.2	0.1	1.7	0.0	384.1	1336.3	2301.4	3960.7
Central-West	Brasília	1470.3	1125.9	0.3	1.8	0.0	382.4	1347.7	2449.4	4414.8
	Campo Grande	1431.8	1019.6	0.4	2.0	0.0	575.1	1267.3	2315.7	4988.4
	Cuiabá	828.9	929.7	1.3	3.6	0.0	131.1	402.2	1346.4	4113.5
	Goiânia	1397.5	1073.3	0.3	1.8	0.0	349.4	1315.0	2324.0	4341.4
Southeast	Belo Horizonte	1433.4	1098.1	0.3	1.9	0.0	398.8	1339.4	2356.0	5246.5
	Rio de Janeiro	1351.6	1155.5	0.5	2.0	0.0	261.1	1100.8	2303.9	4463.2
	São Paulo	1295.5	1126.0	0.6	2.1	0.0	244.6	1013.1	2229.4	3999.1
	Vitória	1382.1	1118.4	0.5	2.0	0.0	323.0	1199.2	2267.8	4193.5
South	Curitiba	1169.1	1013.5	0.7	2.5	0.0	269.1	898.0	1929.0	4101.5
	Florianópolis	1181.8	1076.9	0.7	2.4	0.0	204.9	875.1	1974.3	4184.4
	Porto Alegre	1212.1	1079.5	0.6	2.2	0.0	202.8	954.9	2031.4	3999.9

Table 2S: Descriptive statistics for temperature.

Radiação	Cities	Mean	Std. dev.	Skewness	Kurtosis	Minimum	Q1	Median	Q3	Maximum
North	Belém	28.4	2.9	-0.2	1.9	19.5	25.8	28.8	30.9	37.0
	Boa Vista	28.8	3.5	0.0	1.9	20.5	25.5	29.1	31.8	37.9
	Macapá	27.2	3.0	0.0	2.3	19.5	24.7	27.7	29.4	36.7
	Manaus	28.7	3.1	0.1	2.1	20.6	26.0	28.7	31.1	40.4
	Palmas	29.5	4.2	-0.1	2.3	16.8	26.2	29.8	32.6	41.6
	Porto Velho	27.7	3.8	-0.1	2.3	13.1	24.3	28.2	30.5	38.6
	Rio Branco	26.4	3.6	-0.1	2.9	9.0	23.3	26.5	29.3	38.2
Northeast	Fortaleza	28.5	2.2	-0.5	2.6	20.1	27.1	28.8	30.3	34.4
	João Pessoa	27.7	2.0	-0.5	3.1	19.6	26.3	27.9	29.0	33.1
	Maceió	27.0	2.4	-0.2	2.8	17.2	25.4	27.2	28.8	36.3
	Natal	27.5	1.9	-0.8	3.4	20.3	26.5	27.8	28.8	32.8
	Recife	27.4	2.4	-0.4	2.8	18.0	25.8	27.6	29.2	34.4
	Salvador	26.8	2.5	0.1	2.5	19.2	25.0	26.8	28.6	35.3
	São Luís	28.2	2.2	-0.2	2.4	21.2	26.7	28.4	29.9	41.9
	Teresina	30.0	3.9	-0.1	2.4	16.0	27.0	30.3	32.7	40.8
	Aracaju	27.7	2.1	-0.4	3.0	19.3	26.4	27.9	29.2	35.4
Central-West	Brasília	23.1	3.9	-0.2	2.8	8.7	20.4	23.4	25.9	35.5
	Campo Grande	25.7	5.1	-0.6	3.3	4.0	22.3	26.4	29.5	40.5
	Cuiabá	28.1	4.9	-0.3	3.0	8.7	24.5	28.6	31.5	43.2
	Goiânia	26.1	4.9	-0.5	3.0	6.0	22.7	26.7	29.6	40.5
Southeast	Belo Horizonte	23.6	4.1	-0.2	2.8	7.7	20.8	23.7	26.6	37.8
	Rio de Janeiro	24.4	3.3	0.4	3.0	14.5	22.0	24.1	26.5	38.8
	São Paulo	22.0	4.8	0.0	2.6	4.1	18.5	21.8	25.4	37.3
	Vitória	26.1	3.6	-0.2	2.7	12.6	23.6	26.2	28.6	37.5
South	Curitiba	19.9	4.9	0.0	2.7	-0.2	16.3	19.9	23.3	35.4
	Florianópolis	22.3	4.4	-0.2	3.1	3.3	19.4	22.4	25.4	39.3
	Porto Alegre	21.5	6.0	-0.1	2.7	1.1	17.4	21.7	25.9	40.2

Table 3S: Descriptive statistics for air humidity.

Radiação	Cities	Mean	Std. dev.	Skewness	Kurtosis	Minimum	Q1	Median	Q3	Maximum
North	Belém	74.4	13.4	-0.1	1.9	30.0	63.0	73.0	88.0	100.0
	Boa Vista	64.2	16.7	0.0	2.0	25.0	52.0	63.0	78.0	96.0
	Macapá	83.0	12.1	-1.0	3.6	29.0	76.0	85.0	93.0	100.0
	Manaus	70.3	16.0	-0.1	2.1	23.0	58.0	70.0	84.0	100.0
	Palmas	55.1	21.8	0.0	1.9	10.0	37.0	55.0	73.0	100.0
	Porto Velho	72.9	17.1	-0.4	2.5	15.0	60.6	73.0	89.0	98.0
	Rio Branco	75.9	15.3	-0.9	4.1	10.0	65.7	77.3	90.2	100.0
Northeast	Fortaleza	65.0	12.3	0.3	2.6	29.0	56.0	64.0	73.0	100.0
	João Pessoa	70.5	9.6	0.7	3.0	36.0	64.0	68.7	76.0	95.0
	Maceió	71.5	11.8	0.3	2.3	33.0	63.0	70.0	80.0	100.0
	Natal	73.4	8.8	0.6	3.0	39.0	67.0	72.0	78.0	99.0
	Recife	70.8	12.6	0.3	2.4	28.0	62.0	69.0	79.0	100.0
	Salvador	73.7	10.4	0.1	2.4	42.0	66.0	73.0	81.0	97.0
	São Luís	76.3	11.7	0.1	2.3	32.0	68.0	75.0	85.0	100.0
	Teresina	59.6	20.0	-0.1	2.0	13.0	44.0	60.0	76.0	99.0
	Aracaju	65.7	10.5	0.3	4.2	10.0	59.0	64.7	72.0	100.0
Central-West	Brasília	58.7	21.2	0.0	2.1	10.0	42.0	58.0	75.0	100.0
	Campo Grande	59.6	20.3	-0.1	2.2	10.0	45.0	59.0	76.0	100.0
	Cuiabá	60.2	20.4	-0.3	2.3	8.0	47.5	60.0	77.4	99.0
	Goiânia	55.9	22.7	0.0	1.9	9.0	37.0	55.0	75.0	97.0
Southeast	Belo Horizonte	58.7	17.6	0.0	2.4	10.0	46.0	58.0	72.0	97.0
	Rio de Janeiro	75.2	10.9	-0.9	5.0	13.0	70.0	76.0	83.0	100.0
	São Paulo	63.2	18.8	-0.3	2.3	12.0	49.0	65.0	79.0	100.0
	Vitória	68.0	14.2	0.3	2.4	23.0	58.0	66.6	77.0	100.0
South	Curitiba	66.4	17.1	-0.4	2.5	13.0	55.0	68.0	80.0	100.0
	Florianópolis	73.2	13.0	-0.3	2.7	18.0	64.0	73.0	84.0	97.0
	Porto Alegre	71.1	17.1	-0.2	2.1	18.0	58.0	71.0	86.0	100.0

Table 4S: Descriptive statistics for wind speed.

Radiação	Cities	Mean	Std. dev.	Skewness	Kurtosis	Minimum	Q1	Median	Q3	Maximum
North	Belém	1.1	0.8	0.3	2.8	0.0	0.5	1.1	1.7	7.2
	Boa Vista	2.2	0.9	0.4	3.0	0.1	1.5	2.1	2.7	7.3
	Macapá	1.3	0.7	0.5	3.1	0.1	0.7	1.4	1.8	5.1
	Manaus	1.7	0.8	0.5	3.3	0.1	1.1	1.7	2.2	7.9
	Palmas	2.1	1.6	1.4	5.7	0.0	1.0	1.8	2.7	12.2
	Porto Velho	1.7	0.7	1.0	7.0	0.1	1.0	1.8	2.1	10.2
	Rio Branco	1.6	1.2	0.9	5.0	0.0	0.9	1.7	2.1	10.9
Northeast	Fortaleza	3.2	1.3	-0.3	2.8	0.0	2.3	3.3	4.1	14.6
	João Pessoa	2.5	0.9	0.2	3.4	0.1	2.0	2.6	3.0	7.3
	Maceió	3.6	1.5	-0.3	2.4	0.1	2.6	3.8	4.6	8.5
	Natal	4.7	1.4	0.0	3.1	0.0	3.8	4.8	5.6	10.5
	Recife	2.1	0.9	-0.3	2.6	0.0	1.5	2.2	2.6	5.2
	Salvador	1.6	0.7	0.9	5.8	0.0	1.2	1.6	2.0	6.6
	São Luís	2.4	1.2	-0.3	2.2	0.0	1.5	2.6	3.3	5.7
	Teresina	1.6	1.0	0.5	3.1	0.0	0.7	1.5	2.2	8.7
	Aracaju	2.9	1.5	0.1	2.7	0.0	1.9	3.0	3.9	8.7
Central-West	Brasília	2.7	1.2	0.2	2.9	0.0	1.9	2.7	3.5	10.4
	Campo Grande	3.3	1.6	0.7	4.0	0.0	2.2	3.2	4.2	13.2
	Cuiabá	1.8	0.8	0.9	5.0	0.0	1.3	1.8	2.2	8.4
	Goiânia	1.6	1.1	0.8	3.9	0.0	0.8	1.5	2.3	9.7
Southeast	Belo Horizonte	2.3	1.1	0.2	3.1	0.0	1.5	2.2	3.0	10.2
	Rio de Janeiro	3.0	2.1	1.5	5.7	0.0	1.5	2.4	3.9	17.2
	São Paulo	2.2	1.2	0.3	3.2	0.0	1.4	2.2	3.0	10.9
	Vitória	2.2	1.0	0.4	2.6	0.1	1.4	2.2	2.9	6.7
South	Curitiba	2.3	1.2	0.6	3.8	0.0	1.4	2.2	3.0	9.4
	Florianópolis	2.0	1.6	0.6	2.9	0.0	0.6	1.9	3.0	10.0
	Porto Alegre	1.7	1.0	0.7	3.5	0.0	0.9	1.5	2.3	8.1
